# The Effect of Hormone Types, Concentrations, and Treatment Times on the Rooting Traits of *Morus* ‘Yueshenda 10’ Softwood Cuttings

**DOI:** 10.3390/life13041032

**Published:** 2023-04-17

**Authors:** Jiajia Sun, Hongyun Li, Hanlei Chen, Tiantian Wang, Jin’e Quan, Huitao Bi

**Affiliations:** 1 College of Forest, Henan Agricultural University, Zhengzhou 450002, China; dearperi@163.com (J.S.); 18336253160@163.com (H.C.); wangtiantian04@163.com (T.W.); 2 Management Office, Beijing Tiantan Park, Beijing 100061, China; hyunli926@126.com

**Keywords:** mulberry, softwood cutting, growth regulator, orthogonal experiment, root morphological index

## Abstract

Enhancing the capacity of fruit trees to propagate via cuttings is an important endeavor for the high-quality development of the fruit industry. Optimizing the conditions for the cutting propagation of mulberry seedlings is an important factor that influences the industrial production of this plant; however, the currently used mulberry breeding technology system is not mature. In this experiment, an orthogonal design was used to intercept semi-woody shoots of Yueshenda 10 as cuttings and set different hormone concentrations (200, 500, 800, and 1000 mg/L), different hormone types (NAA, IBA, IAA, and ABT-1), and different soaking times (10, 30, 60, and 120 min) for cuttings. The effects of the three factors on the rooting of mulberry cuttings were investigated by soaking the cuttings in clean water for 10 min as a control. The results showed that the primary and secondary order of the three factors affecting the rooting rate of cuttings was hormone concentration > hormone type > soaking time, and the concentration of exogenous hormones had a significant impact on all rooting indicators (*p* < 0.05). In addition, the rooting rate (66.24%), average number of roots (7.54 roots/plant), and rooting effect index (4.23) of Yueshenda 10 cuttings reached the optimal level when soaked with 800 mg/L ABT-1 for 30 min. The longest root length (10.20 cm) and average root length (4.44 cm) of cuttings achieved the best results when soaked with 800 mg/L NAA for 60 min and 500 mg/L NAA for 30 min, respectively. On balance, it is considered that the preferred solution is to soak the cuttings of Yueshenda 10 with 800 mg/L ABT1 solution for 0.5 h.

## 1. Introduction

Mulberry *(Morus*) belongs to the family Moraceae and is a perennial woody plant. The fruit mulberry is a type of mulberry tree chosen from the Moraceae family of plants specifically for producing appropriate mulberries for either direct consumption or processing [1]. The production, life, and ecological functions of mulberry fruits, leaves, and branches make fruit mulberry not only an economically valuable forest tree but also a tree species with significant value for environmental management and ecological optimization [2].

The need for healthy, green, and efficient development of the fruit mulberry industry calls for improvement of the breeding and cultivation ability, starting with the provision of sufficient high-quality seedlings. Currently, there are three commonly used asexual reproduction techniques for mulberry seedling cultivation: tissue culture, grafting, and cutting [3]. When compared to tissue culture, which has high prerequisites, and grafting, which has a lengthy cycle [4], cutting propagation, particularly softwood cutting, can propagate substantial quantities while retaining the superior characteristics of the parent plant and preventing deterioration. Furthermore, it is straightforward to manage and promote at a lower cost [5,6,7]. Therefore, cutting propagation has become one of the main seedling breeding methods that is ideal for the large-scale planting needs of mulberry orchards [8,9,10].

Investigations into the effects of plant growth regulators on the rooting of cuttings dates back to 1934 when Went explored the types, concentrations, and treatment times of exogenous hormones [11,12]. The formation of adventitious roots is a process regulated by various factors, including the lignification degree of the cutting, the season of cutting, the rooting environment, and exogenous hormones [13]. Among these factors, the cutting medium and hormone application are two essential external factors affecting the rooting and root quality of cuttings [14].

In the 1930s, indole-3-acetic acid (IAA) was proven to be effective in promoting the formation of adventitious root [15]. Indole-3-butyric acid (IBA) has become the most commonly used natural auxin for plant reproduction in horticulture and forestry because of its stability and effectiveness in promoting rooting of stem cuttings [16,17]. A-naphthylacetic acid (NAA) plays an important role in promoting cell division and expansion, inducing adventitious root formation, increasing fruit setting, and preventing fruit drop [18]. Different phytohormone concentrations also result in different plant growth, development, and responses to external stimuli [19,20]. 

Currently, these plant growth regulators are widely used in cutting seedling cultivation. ABT1 and ABT2 rooting powders have been reported to increase rooting rates up to 95% in green stem cuttings [21]. For *Morus alba* L., the application of 0.10% to 0.13% IBA and ABT rooting powder No. 6 resulted in a survival rate of over 81% [22,23,24]. Other studies have demonstrated that the combination of NAA and IAA may be better compared with NAA alone in the propagation of mulberry [25,26]. Deng et al. compared the effects of IAA-3-acetic acid and ABT1 rooting powder alone and in combination and found that the combination of the two had more benefits compare to the use of either of the two alone [27].

These studies have demonstrated that auxins can promote the growth of adventitious rooting of cuttings. The aim of this study was to determine the most suitable hormone combination for softwood cutting propagation of mulberry to provide a basis for developing high-quality mulberry seedlings within a short time to meet the high market demand. The results are expected to provide a theoretical basis and technical guidance for the propagation of mulberry through softwood cutting. Therefore, a green stem cutting experiment was conducted on Yueshenda 10, and the effects of different influencing factors on the rooting characteristics of Yueshenda 10 softwood cuttings were compared using an orthogonal experimental design based on previous studies. 

Therefore, this study aims to explore the most suitable hormone combinations for mulberry shoot cutting based on existing research in order to breed more excellent mulberry seedlings in a short period of time, meet market production and market demand, and provide a theoretical basis and technical guidance for mulberry shoot cutting propagation. To this end, we conducted a green branch cutting experiment on Yueshenda 10. By applying orthogonal experimental design, we compared the effects of different influencing factors on the rooting characteristics of Yueshenda 10 during the tender branch cutting process.

## 2. Materials and Methods

### 2.1. Materials

The experiment was conducted in the third residential area of Henan Agricultural University in Zhengzhou City, Henan Province. Henan Province is the birthplace of silkworm production, which includes subtropical and warm temperate regions with an average annual temperature of 12~15 °C, annual rainfall of 600~1200 mm, and 2000~2600 h of sunshine per year. The region has a large temperature difference between day and night [28]. The third residential area of Henan Agricultural University is located at 113.22° E and 34.28° N and is equipped with a fully-illuminated automatic spray greenhouse with a propagation pool measuring 11 m in length, 6 m in width, and 0.4 m in depth. The pool is evenly divided into five small propagation pools, each with a length of 6 m, width of 2 m, and depth of 0.4 m.

The mulberry cuttings used for experiments were obtained from 3-year-old mulberry trees in the experimental mulberry orchard located in the third residential area of Henan Agricultural University. “Yueshenda 10” (*Morus atropurpurea* Roxb. ‘Yueshenda 10’) is a fruit mulberry variety selected by the Institute of Sericulture and Agroprocessing, Guangzhou Academy of Agricultural Sciences, Guangdong Province, China. This germplasm was introduced and preserved in the ex situ conservation bank of mulberry germplasm resources of Henan Agricultural University. The test material was selected from 3-year-old live seedlings of “Yueshenda 10” in the mulberry garden of the third living area of Henan Agricultural University. 

The cuttings were made from semi-lignified branches, measuring 15 cm long with two to three semi-leaves (retaining half of the leaf blade), a flat cut on the upper end, and a 45° oblique cut on the lower end. The indole-3-acetic acid (IAA), indole-3-butyric acid (IBA), naphthaleneacetic acid (NAA), and ABT1 rooting powder used in this study were all purchased from Solabio Technology Co., Ltd. in Beijing, China. The growth factors used in this experiment were indole-3-butyric acid (IBA), indole-3-acetic acid (IAA), and naphthalene acetic acid (NAA) produced by Beijing Solarbio Science & Technology Co., Ltd., Beijing, China. Rooting powder (ABT1) was produced by the Forestry Research Institute of China Academy of Forestry Science. The propagation substrate was river sand purchased from the market.

### 2.2. Experimental Design

We designed an experiment to investigate the effects of plant growth hormones, the concentration, and the immersion time on the rooting of mulberry cuttings. This experiment adopts an orthogonal design with three factors: the exogenous hormone concentration type, exogenous hormone concentration, and soaking time, represented by A, B, and C. Each factor has four levels as shown in Table 1. For each factor, the tests were performed at four levels as shown in Table 1. The L16(4^3^) orthogonal experimental design was employed (Table 2) with 17 treatments, including a control group (CK). Each treatment was replicated three times, and 150 cuttings were used in each repeat. Using the orthogonal experimental design of L16 (43) (Table 2), a total of 17 treatments were added with water treatment (CK). Each treatment was repeated three times, with 50 cuttings per repetition, resulting in a total of 2550 cuttings.

Initially, the soil in each cutting pot was disinfected by spraying with a 1:400 dilution of carbendazim, turned over at least three times, and dried for 3 days. On the day of cutting, the cuttings were dipped in a 5% water solution of carbendazim for 10 s, while cuttings for the CK group were treated with water. The cuttings were then inserted into the pots according to the experimental design. After cutting, an automatic spray device was used to enhance the moisture and maintain the relative humidity of the air at 70% to 80%. Before cutting, according to a 1:400 dilution of carbendazim, spray application for each cutting pool was performed three times for soil disinfection, and the samples were dried for 3 days. On the morning of the cutting day, cuttings were cut from a well-growing and pest-free mother tree and immediately treated. 

First, we used 50% carbendazim water at 800 times liquid speed for 10 s. Secondly, we prepared solutions for orthogonal experiments. The method is as follows: We used a balance to weigh 500 mg of IAA. First, we dissolved 500 mg of IAA in a small amount of 75% ethanol, and then we added 1 L of water to prepare a 500 mg · L^−1^ IAA solution. We prepared IAA solutions of other concentrations and IBA, NAA, and ABT1 solutions in the same way. Finally, we placed the cuttings in the prepared plant growth regulator solution and slowly soaked them according to the experimental arrangement (Table 2). After cutting, the humidity was increased by automatic intermittent spraying facilities arranged in the shed to maintain the relative humidity of the air at 70–80%.

### 2.3. Measurement of Indicators

Three stems with similar stages of development were collected from each treatment group on days 1, 10, 20, 30, and 40 after cutting, and 2 cm of the bark was promptly sliced and blended with scissors. This process was repeated three times, and the samples were wrapped in tin foil, stored briefly in liquid nitrogen, and then stored at −80 °C until further measurement.

Analysis of rooting traits: at 50 days after cutting, several rooting traits, including the rooting rate, number of roots, average root number, average root length, longest root length, and rooting effect index [29], were evaluated for the 17 treatments. The formulae for these indicators are as follows:

Rooting rate = Number of cuttings rooted/Total number of cuttings × 100%

Average root number = Total root number of cuttings/Number of rooted cuttings

Average root length = Total root length of cuttings/Number of roots

Rooting effect index = (Average root length × Number of rooted cuttings)/Total number of cuttings.

### 2.4. Data Analysis

Data preprocessing and statistical analysis were conducted using Excel 2003. Range analysis was performed to preliminarily identify the optimal combination of treatments. Multiple-factor variance analysis was performed using SPSS 24.0 to determine the influence of each factor on the experimental results, and the least significant difference method (LSD) was employed to conduct multiple comparisons. The membership function method was applied to perform comprehensive evaluation of the rooting and propagation effects of each treatment combination. We drew the chart using Origin 2021.

The membership function calculation method was as follows: U(Xj) = (Xj − Xmin)/(Xmax − Xmin). The membership values of different indicators for each treatment were summed, and the average value was ranked. The larger the average value, the better the rooting effect.

## 3. Results and Analysis

### 3.1. Rooting Types and Process of Cutting Propagation

**Figure 1:**
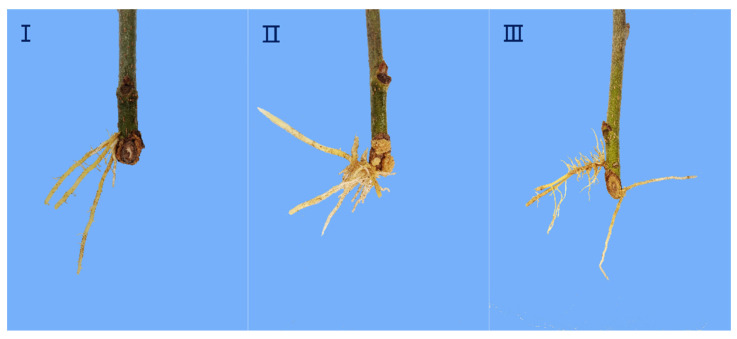
The rooting type of cuttings. (**I**) the root epidermis rooting, (**II**) the callus rooting, and (**III**) mixed rooting.

**Figure 2:**
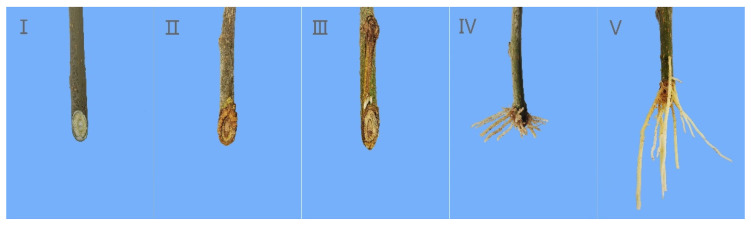
The rooting process of cuttings. Sorted according to the rooting process of cuttings (**I**–**V**).

The rooting types of softwood cuttings were observed after planting, and the results showed that the main rooting type was callus formation (type II), followed by epidermal rooting (type I), and few roots were formed at the junction of callus and epidermis, which was classified as the mixed rooting (type III) (see Figure 1). The rooting process of cuttings was divided into five stages (see Figure 2): the callus formation stage (stage II) from 0 to 10 days after planting, the induction stage of root primordium (stage III) from 10 to 20 days, the expression and formation stage of adventitious roots (stage IV) from 20 to 30 days, and the elongation and development stage of adventitious roots (stage V) after 40 days. The control treatment resulted in the delayed formation of callus and adventitious root formation and had a lower rooting rate compared to the experimental treatments.

### 3.2. Analysis of Primary and Secondary Effects of Different Factors

**Figure 3:**
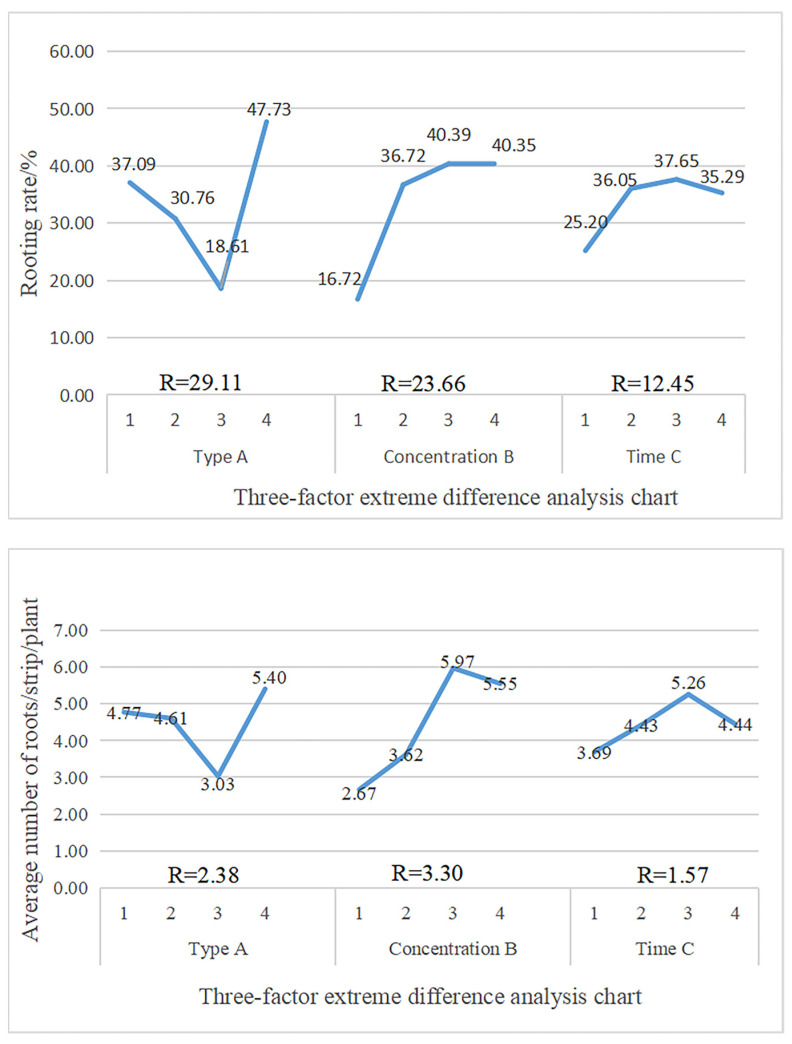

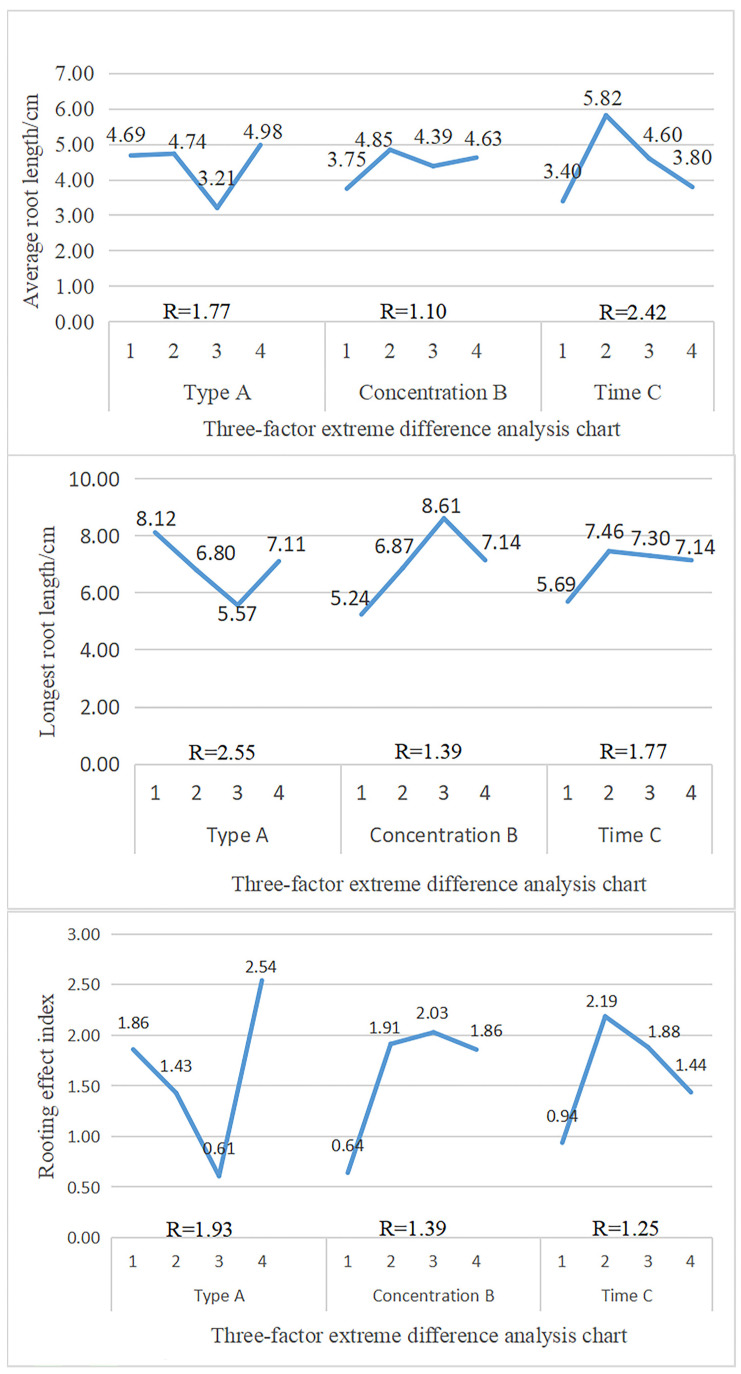
Range analysis for the influence of various test factors on the rooting index.

Figure 3 demonstrates that if the rooting rate is only taken as the primary indicator, the rooting rates for different types, concentrations, and treatment times of hormones were 29.11, 23.66, and 12.45, respectively. This demonstrates that the main factors affecting the softwood cutting rooting of mulberry were hormone type > hormone concentration > treatment time. 

The values of K1, K2, and K3 indicated that the treatment combination with the best rooting promotion effect was the slow immersion of the cuttings in 800 mg/L ABT1 solution for 60 min (A4B3C3) with the highest rooting rate of 66.24%. When only the average root number was considered as the primary indicator, from the R-value of the range, it can be inferred that hormone concentration (3.30) > hormone type (2.38) > treatment time (1.57) had the least influence on the average root number, implying that hormone concentration had the strongest effect, and treatment time had the least effect. 

The K values indicate that the theoretical optimal combination is to soak the cuttings with 800 mg/L ABT1 for 60 min (A4B3C3). Considering only the indicator of average root length, the K1, K2, and K3 values of each factor indicated that the longest average root length of fruit mulberry could be obtained by soaking 500 mg/L ABT1 for 30 min (A4B2C2). 

The R-values influencing the average root length of cuttings were treatment time (2.42) > hormone type (1.77) > hormone concentration (1.10), which suggests that treatment time was the most important factor influencing the average root length, whereas hormone concentration showed the smallest effect. If the longest root length was taken as the primary indicator, we observed that the order of influence on the longest root length of cuttings was hormone concentration > hormone type > treatment time, indicating that concentration (3.37) was the most important factor influencing the longest root length of softwood cuttings, whereas hormone type (2.55) and treatment time (1.77) had the least effects. 

Based on the K1, K2, and K3 values of the three factors, the optimal combination of plant growth regulators was determined to be soaking cuttings with 800 mg/L NAA for 30 min (A1B3C2). Taking only the rooting effect index as the primary indicator, the R-value showed that the main factor influencing the rooting effect index of green shoot cuttings was the hormone type (1.93) > hormone concentration (1.39) > treatment time (1.25). In addition, analysis of the K1, K2, and K3 values of each factor revealed that A4B3C2 was the best combination for the mulberry softwood cutting rooting effect index, i.e., soaking in an 800 mg/L ABT1 solution for 30 min achieved the best rooting effect index of 4.23.

In terms of the rooting rate and average number of roots, soaking cuttings with 800 mg/L ABT1 for 30 min (A4B3C3) was the best combination. However, the other three theoretically optimal combinations were not observed in the experiment. It remains to be further tested whether the effectiveness of these combinations is influenced by the interaction of factor levels.

### 3.3. Effects of Hormone Types, Concentrations, and Soaking Time on Rooting Indicators of Yueshenda 10 Cuttings

Five indicators, including the rooting rate, average number of roots, average root length, longest root length, and rooting index, were used to evaluate the rooting effect of Yueshenda 10 green cuttings. The results were subjected to variance analysis (Table 3) and LSD multiple comparison tests (Figure 3) as shown in the table below.

#### 3.3.1. Effects of Factors and Their Interactions on Rooting Indicators of Cuttings

Visual and straightforward representation of the experimental results can be achieved through range analysis; however, this does not convey information about the magnitude and precision of errors. Therefore, variance analysis should be performed [30]. The results of three-factor variance analysis (Table 3) showed that the concentration of growth regulators had the greatest impact on the rooting of cuttings (*p* < 0.05), except for the average root length. This concentration also had a significant effect on the rooting rate, average number of roots, longest root length, and rooting index (*p* < 0.01). 

Hormone type caused a secondary effect and significantly affected the rooting rate and rooting index. The effect of the treatment time on rooting indicators was relatively small, only significantly affecting the longest root length, that is, hormone concentration > hormone type > treatment time. In terms of interaction among two factors, the interaction between the hormone concentration and treatment time had the greatest impact on rooting with significant and highly significant effects on the rooting rate and rooting index, respectively. 

The interaction between hormone type and treatment time had a smaller effect, only significantly affecting the longest root length. The interaction effect between hormone type and concentration had no significant effect on any tested parameter. A comprehensive analysis concluded that the interaction effect between hormone concentration and treatment time was the main factor affecting the rooting of mulberry green cuttings.

#### 3.3.2. Effects of Hormone Types on Rooting Index of Yueshenda 10 Cuttings

**Figure 4:**
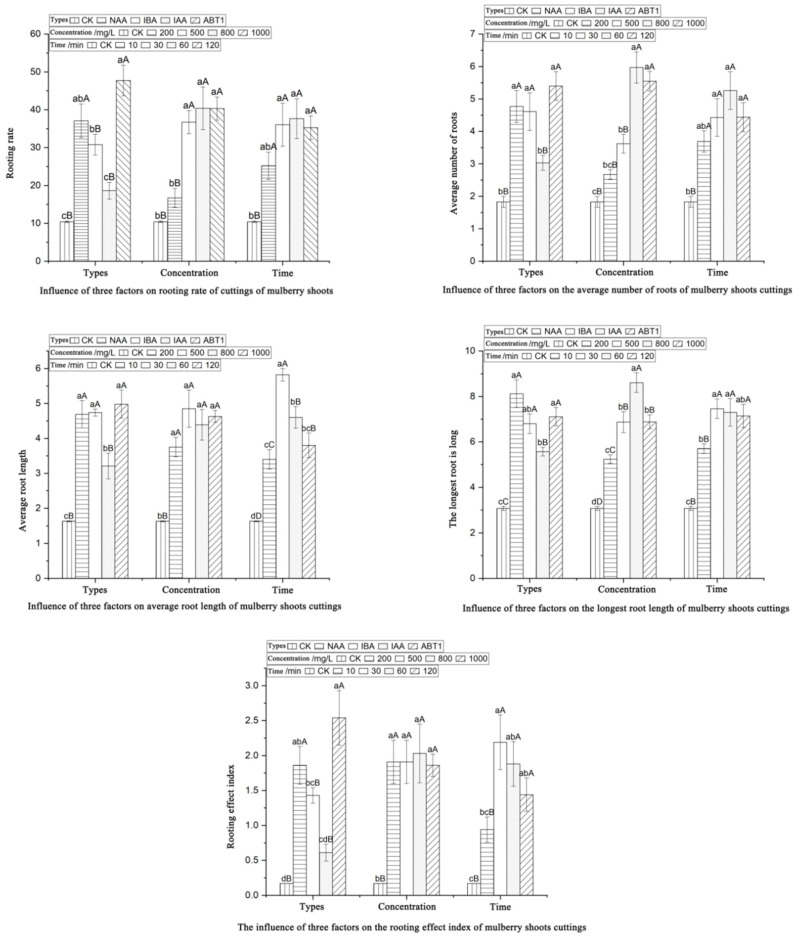
LSD multiple comparisons of the effects of the hormone type, hormone concentration, and time on the cutting rooting parameters of Yueshenda 10. Under each influencing factor, different uppercase letters indicate extremely significant differences between treatments (*p* < 0.01), while different lowercase letters indicate significant differences between treatments (*p* < 0.05).

The data presented in Table 3 suggest that the significant probabilities (*p* values) of the rooting rate and rooting effect index among the five rooting indices of plant growth regulators were both below 0.05, indicating significant differences in the rooting rate and rooting effect index between hormone types. However, the differences in the other three rooting indices were not significant. Figure 4 also illustrates that the rooting rate, average root number, and rooting effect index were consistent among the four plant growth regulators and CK (distilled water) treatments. 

The ranking of the five rooting indices from best to worst was as follows: ABT1, NAA, IBA, IAA, and CK. ABT1 treatment was significantly or extremely significantly different from other hormones, with ABT1 and NAA showing superior performance compared to IAA and CK. The rooting rate (47.73%), average root number (5.40 per plant), average root length (4.98 cm), and rooting effect index (2.54) under ABT1 treatment were the best, whereas those of NAA, IBA, and IAA were the worst.

#### 3.3.3. Effects of Concentration on Rooting Index of Cuttings

As shown in Table 3, the F-values of the variance analysis results of different concentrations for each index are larger than the critical value, indicating significant differences. According to the multiple comparison results shown in Figure 4, the rooting rate, average number of roots, and longest root length exhibited similar responses to the four concentration levels and the CK (control) treatments. The optimal treatment was observed at a concentration of 800 mg/L, followed by concentrations of 1000 and 500 mg/L, whereas concentrations of 200 mg/L and CK were the least effective. 

The longest average root length was observed at a concentration of 500 mg/L, and there were no significant differences between the four concentration levels and the CK treatment at the 0.05 and 0.01 significance levels, but all were significantly higher compared with the values for CK. The rooting rate and rooting effect index revealed were not significantly different among concentration levels 2, 3, and 4 but were significantly higher than levels 1 and the control treatment. The rooting rate (40.39%), average number of roots (5.97 per plant), longest root length (8.61 cm), and rooting effect index (2.03) were all optimal at a hormone concentration of 800 mg/L.

#### 3.3.4. Effects of Treatment Time on Rooting Indexes of Stem Cuttings

The rooting rate (37.65%) and mean root number (5.26 cm) were the highest after 60 min of treatment. At the three treatment durations of 30, 60, and 120 min, the above parameters were not significantly altered at the 0.05 and 0.01 levels but were significantly higher than the 10 min and water treatments with extremely significant differences compared to the water treatment. The mean root length, maximum root length, and rooting effectiveness index showed consistent results among the four treatment durations and the water treatment, with the rooting effectiveness rankings being 30 min, 60 min, 120 min, 10 min, and water treatment, in that order. 

The mean root length (5.82 cm), maximum root length (7.46 cm), and rooting effectiveness index (2.19) were optimal at the 30 min treatment duration. Significant differences were recorded in the mean root length among different soaking times, with the 30 min treatment being extremely significant higher compared with the levels at the 60 and 120 min treatments, and levels 3 and 4 being extremely significantly higher than the 10 min and water treatments. The maximum root lengths of the 30 and 60 min treatments were not significantly different at the 0.05 and 0.01 levels but were significantly higher than the 120 min treatment and were extremely significantly higher relative to the other treatment levels. 

The rooting effect index showed no significant difference between the 60 min and 120 min treatments (*p* > 0.05), but the effect between the 60 min and 120 min treatments and the 10 min and clear water treatments was extremely significant (*p* < 0.01). 

### 3.4. Fuzzy Function Analysis of the Optimal Combination of Hormone Type, Concentration, and Soaking Time for the Rooting of Yueshenda 10 Stem Cuttings

The study discovered notable variations in the four rooting indicators across the 16 treatment combinations. The various rooting indicators did not exhibit identical levels of effectiveness within the same treatment combination. Therefore, using the membership function method, a comprehensive analysis was conducted on the five rooting indicators, including the rooting rate, average root number, average root length, longest root length, and rooting effect index, under the 16 treatment combinations. The ranking of each treatment U(Xj) is shown in Table 4. 

The results showed that, among the top five treatments in terms of the overall cutting effect U (Xj), the main types of exogenous hormones were ABT1 and NAA, and the rooting rates of the top four combinations were also in the top four. It can be seen that hormone types have a significant impact on the rooting rate of cuttings, which is consistent with the results of the analysis of variance. The membership function value of the three-factor treatment combination 15 was the highest, i.e., the rooting effect of cuttings was optimal under the A4B3C2 treatment, which is consistent with the optimal combination analysis of the rooting effect index. 

The rooting rate (66.24%), average root number (7.54), and rooting effect index (0.96) were ranked the best, whereas other indicators ranked were ranked among the top four. In other words, the best softwood cutting effect of Yueshenda 10 was achieved following treatment of ABT1 at a concentration of 800 mg/L and a treatment time of 30 min. The combination of soaking with 500 mg/L ABT1 for 60 min was the second best, with the rooting rate (52.95%), average root length (6.23 cm), and rooting effect index (3.30) being ranked among the top three. 

Although the rooting rate (47.29%) of the combination of soaking with 800 mg/L NAA for 60 min was not ranked among the top three, its longest root length (10.30 cm) and average root number (6.89) both showed excellent performance, making it ideal for the cultivation of high-quality seedlings with developed root systems in areas with abundant cutting materials.

## 4. Discussion

Root cuttings can be divided into three types: epidermal rooting [31], callus rooting [32,33], and combined rooting, with the number and proportion of roots in each part being the main classification criterion. In this study, basal morphological analysis of rooting cuttings revealed that the majority of roots of the Yueshenda 10 tender shoot cuttings originated from callus tissue, whereas a small number of cuttings developed adventitious roots originating from the epidermis, and very few had roots originating from both sites. 

This is consistent with the results reported by Shen et al. [34] for mulberry cutting seedlings, indicating that softwood cuttings of fruit mulberry belong to the callus rooting type, and that fruit mulberry is a difficult-to-root species. In addition, by observing the growth of cuttings’ roots, it can be seen that the devel-opment of adventitious roots of mulberry branches went through five main stages. Next, the anatomical structure of roots is studied to confirm whether this judgment is sufficiently correct.

The process of cutting propagation is not only affected by the genetic characteristics, ecology, and biology but is also modulated by several external factors, such as light, air, temperature, and humidity [35]. Many studies have shown that exogenous plant hormones can promote the formation of adventitious roots in cuttings and can induce rooting in plants that are difficult to root. Different types of exogenous hormones promote the formation of adventitious root to different degrees [36,37,38]. In this study, the effects of different types of growth regulators, different concentrations, and treatment times, as well as their interactions on the rooting of softwood cuttings of *Morus alba* L., were comprehensively explored.

### 4.1. Effects of Plant Growth Regulators on Mulberry Cuttings

Currently, the hormones commonly used in production include ABT rooting powder, indole-3-acetic acid (IAA), indole-3-butyric acid (IBA), naphthylacetic acid (NAA), naphthaleneacetic acid amide, and other benzoxycarboxylic acid compounds. In this study, all plant growth regulators used promoted the rooting of softwood cuttings of mulberry. 

This may be because exogenous IBA can potentially enhance the nutritional level of the rooting zone of trees, the content of endogenous hormones, and the activity of peroxidase, thus, providing suitable conditions for the formation of adventitious roots and increasing rooting rate and root growth [39,40,41]. ABT can not only potentiate the effects of exogenous hormones and rooting substances needed for cutting rooting but also can promote the synthesis of endogenous auxin, which accelerates the healing of the lower cutting surface and promotes rooting [42]. Some studies have also reported that NAA is a potential treatment for plants that show an impaired ability to root [43,44].

Significant research has shown that ABT No. 1 is suitable for plants with high economic value and that are difficult to root [45]. It has been found that ABT No. 1 is more effective than NAA and IBA in rooting studies of Catalpa bungei [46], wild European plum [47] and Ulmus macrocarpa [48]. There is also evidence that IAA is less effective than IBA and NAA in promoting cutting rooting [49,50]. Geng Wenjuan et al. [47] reported that the survival rate of cuttings treated with 800 mg/L ABT rooting powder was as high as 80.00%, and the rooting rate was as high as 76.67%. 

In this study, NAA, IBA, IAA, and ABT1 were used to treat cuttings at four levels, and the results showed that ABT1 had the best seedling growth effect with the highest rooting rate, average number of roots, average root length, and rooting effect index. In comparison, NAA and IBA were less effective than ABT1, whereas IAA had the poorest effect. The effects of the four growth regulators on the seedling growth were consistent with those reported previously. 

In addition, evidence from similar studies has shown that the effect of cutting rootings under mixed treatment with plant growth regulators is better than that of using a single growth regulator [22,51]. For example, when using a mixture of NAA and IBA to treat softwood cuttings, the mixed treatment showed better outcomes compared with NAA or IBA alone [52]. However, the combined use of growth regulators was not explored in this study and, thus, needs to be further investigated.

### 4.2. Effects of Plant Growth Regulator Concentration on the Rooting of Mulberry Cuttings

Plant growth regulator (PGR) concentration is one of the main factors affecting the rooting of plant cuttings. Appropriate concentrations can improve the rooting and root growth, while excessively high concentrations can damage the cutting tissues and impair the rooting process [53,54]. 

Plant growth regulator concentrations showed significant effects (*p* < 0.05) on the rooting of hairy rosemary [55] and hops [56] plug cuttings in rooting studies. Meng Haisan et al. [57] concluded that the effect of exogenous hormone concentrations on the rooting rate, mean root length, and mean root number of blueberry cuttings reached significant levels (*p* < 0.05). In addition, the main factor affecting the root length of baldcypress cuttings was also the hormone mass concentration [58]. Our results are consistent with those of previous studies, suggesting that the PGR concentration significantly affects all the tested indicators. Wang Bangqin et al. found that, with an ABT1 soaking time of 0.5 h, the rooting rate of cuttings increased with the PGR concentration, reaching a peak before decreasing [59]. 

Luo Xuemei et al. [60] and Xu Yin et al. [61] reported that, in softwood cutting experiments, ABT rooting powder had the best overall cutting performance at a concentration of 800 mg/L, which is consistent with the results of this study.

Furthermore, this study observed a relatively uniform pattern of response from the different indicators to varying levels of PGR concentration. As the concentration of PGR quality increased, the rooting rate, average number of roots, maximum root length, and rooting efficiency index all demonstrated an initial increase followed by a subsequent decrease. As the concentration gradually increased within the range of 0~200~500~800 mg/L, it began to decrease at 1000 mg/L. The optimal treatment was at 800 mg/L, and within a certain range, the higher the quality concentration, the better the rooting effect, but excessive PGR concentration can inhibit rooting. 

In contrast, Rovier, V. et al. concluded, in a study on Brazilian native medicinal plants, that the application of high concentrations of IBA could better promote the root development of the spike with a maximum rooting rate of 79.17% [62]. The differences between the results of this study and theirs may be due to differences in the variety, genetics, and physiological conditions of the cuttings or may be related to the experimental design.

### 4.3. Effects of Treatment Time on the Propagation of Mulberry Cuttings

The soaking time of plant growth regulators can affect the propagation index of cuttings. Cheng et al. found that the rooting rate of softwood cuttings in maple was the highest when soaked in NAA or ABT for 30 min [63]. Chen et al. demonstrated that soaking time significantly affected the survival rate, total number, and length of roots in Betula albosinensis cuttings with the best results obtained after a 2 h soaking period [64]. 

In the study, soaking time only had a significant effect on the longest root length of tender mulberry cuttings, and it had no significant effects on the rooting rate, average number of roots, average root length, and rooting effectiveness index, which is inconsistent with the results by Chen et al. This discrepancy may be related to the plant’s own genetic factors, physiological conditions, and environmental conditions during propagation.

The results of the orthogonal test conducted revealed that the best treatment approach for enhancing the rooting rate of delicate mulberry softwood cuttings was immersing them in 800 mg/L ABT1 for 30 min. However, this particular approach was not deemed the most effective for increasing the average or maximum length of the roots. In the actual reproduction production of “Yueshenda 10”, different combinations of plant growth regulators can be selected based on the desired propagation index. For example, if a longer average root length is required, cuttings can be soaked in 500 mg/L NAA for 30 min, while if a longer longest root length is desired, cuttings can be soaked in 800 mg/L NAA for 1 h.

### 4.4. Effects of Interaction on the Propagation of Mulberry Cuttings

Numerous studies have shown that the hormone type, concentration, and time not only individually affect the rooting of cuttings but also have significant interactive effects on each other [65,66]. Quan et al. reported that the concentration and soaking time of IBA, as well as their interaction, significantly influenced the rooting rate, number of roots, root length, and rooting index of cuttings [67]. Shen et al. discovered that different concentrations and soaking times of NAA had significant effects on cutting rooting, and the interaction between different concentrations of NAA and soaking time also affected the rooting [68]. 

Moreover, the interaction between different concentrations of ABT1 and soaking time showed strong modulatory effects on the rooting of semi-lignified shoots in the current year. However, Jussara et al. found that the interaction between hormone types and treatment time did not significantly affect the rooting of cuttings [69]. In this study, we found that the interaction between hormone concentration and treatment time significantly and extremely significantly affected the rooting rate and rooting index of cuttings, respectively.

Yi et al. found that the interaction effect of the rooting hormone and soaking time had an extremely significant effect on the rooting of stem cuttings of Cyclocarya paliurus [70]; Hu et al. found that the interaction effects of hormone type × hormone concentration, hormone type × soaking time, and three-factor interaction significantly affected the rooting percentage and longest root length of Picea abies cuttings [71]. 

Our results show that the interaction effects of hormone type × treatment time mainly influenced the longest root length of the cuttings, while the three-factor interaction only had a significant effect on the average root length. Moreover, none of the interactive effects influenced the average number of roots. These results are consistent with the findings by Ou et al., who reported that the interaction effect between rooting promoters and concentration did not affect the rooting percentage, average number of roots, and total root length of cuttings [72], and this was different from the findings by Yi et al. [70].

## 5. Conclusions

This study indicates that the Yueshenda 10 mulberry cultivar belongs to the type of callus rootings, and its softwood cuttings undergo five developmental stages during the propagation process. Among the factors affecting the softwood cuttings, hormone concentration was the key factor, followed by hormone type, while soaking time had little effect. In addition, the optimal treatment combination for softwood cuttings (800 mg/L ABT1, soaking for 30 min, and using river sand as the rooting substrate) was determined, which achieved a rooting rate of 66.24% and effectively solved the practical problem of difficult rooting in this cultivar of mulberry. These treatment settings can, therefore, be applied in large-scale seedling cultivation.

Exogenous auxin treatment altered the growth level and interrelationships among the five rooting indicators during the rooting process of Yueshenda 10 softwood cuttings, which indicated specific changes and was closely related to the rooting initiation and development. However, the rooting of softwood cuttings is an extremely complex physiological process, and subsequent research based on this study will focus on the composite physiological and biochemical characteristics and genetic traits of mulberry seedlings to further reveal the rooting mechanism of Yueshenda 10 softwood cuttings and clarify the mechanisms underlying the mulberry rooting process.

The exogenous auxin treatment changed the growth level and relationship of five rooting indicators in the rooting process of “Yueshendashi” cuttings, which showed a certain regularity in the rooting process and was closely related to the occurrence and development of cuttings rooting. This experiment found that hormone concentration was the key factor affecting the rooting changes of cuttings, followed by hormone type, and treatment time had little effect. 

In addition, the optimal treatment combination for softwood cutting was determined to be soaking the cuttings in 800 mg/L ABT1 for 30 min using river sand as the rooting medium, and the highest rooting rate reached 66.24%. This treatment effectively solves the problem of the actual rooting difficulties for this variety of mulberry tree and can be considered for large-scale seedling cultivation. In the future, we will further study the molecular mechanism of adventitious roots of mulberry in terms of auxin regulation.

## Figures and Tables

**Table 1:** Test factors and levels.

Experimental Level	Experimental Factors		
	Types of Hormones(A)	Concentration/mg/L(B)	Soaking time/Min(C)
1	NAA(A1)	200(B1)	10(C1)
2	IBA(A2)	500(B2)	30(C2)
3	IAA(A3)	800(B3)	60(C3)
4	ABT1(A4)	1000(B4)	120(C4)

**Table 2:** L16(4^3^) three-factor four-level orthogonal experimental design.

Treatment Number	Treatment Combination	Types of Hormones(A)	Concentration/mg/L(B)	Soaking Time/Min(C)
1	A1B1C1	NAA(A1)	200(B1)	10(C1)
2	A1B2C2	NAA(A1)	500(B2)	30(C2)
3	A1B3C3	NAA(A1)	800(B3)	60(C3)
4	A1B4C4	NAA(A1)	1000(B4)	120(C4)
5	A2B1C2	IBA(A2)	200(B1)	30(C2)
6	A2B2C1	IBA(A2)	500(B2)	10(C1)
7	A2B3C4	IBA(A2)	800(B3)	120(C4)
8	A2B4C3	IBA(A2)	1000(B4)	60(C3)
9	A3B1C3	IAA(A3)	200(B1)	60(C3)
10	A3B2C4	IAA(A3)	500(B2)	120(C4)
11	A3B3C1	IAA(A3)	800(B3)	10(C1)
12	A3B4C2	IAA(A3)	1000(B4)	30(C2)
13	A4B1C4	ABT1(A4)	200(B1)	120(C4)
14	A4B2C3	ABT1(A4)	500(B2)	60(C3)
15	A4B3C2	ABT1(A4)	800(B3)	30(C2)
16	A4B4C1	ABT1(A4)	1000(B4)	10(C1)
17	Control group (CK)	Water	0	10

**Table 3:** Correlation analysis between different hormone types, concentrations, and treatment times and changes in the rooting index of cuttings (* *p* < 0.05 and ** *p* < 0.01).

Source of Error	Rooting Rate		Average Number of Roots		Average Root Length		Longest Root Length		Rooting Index	
	F	*p*	F	*p*	F	*p*	F	*p*	F	*p*
A	6.245 *	0.016	3.730	0.060	1.977	0.165	0.016	0.901	4.555 *	0.039
B	24.746 **	0.000	59.980 **	0.000	7.157 *	0.011	32.649 **	0.000	12.957 **	0.001
C	3.070	0.087	3.034	0.089	0.978	0.328	11.926 **	0.001	1.035	0.315
A × B	0.000	0.983	0.829	0.368	3.415	0.072	0.996	0.324	0.165	0.687
A × C	0.172	0.680	0.016	0.900	1.079	0.305	6.899 *	0.012	0.004	0.950
B × C	5.706 *	0.021	3.272	0.076	1.523	0.024	0.011	0.917	8.492 **	0.006
A × B × C	0.000	0.997	0.264	0.610	7.060 *	0.011	2.222	0.143	1.496	0.228

Note: A: hormone type; B: Concentration; and C: Processing time. The “*” shows significant differences between each treatment while *p* < 0.05, and the “**” shows very significant differences between each treatment while *p* < 0.01. Data on the rooting rate were squared, and then the arcsine was subjected to analysis of variance.

**Table 4:** Effects of different factor combinations on indexes of mulberry softwood cutting and evaluation of the membership function.

Treatment Combination		Rooting Rate (%)	Average Root Number (Root/Plant)	Average Root Length/cm	Length of Longest Root/cm	Rooting Effect Index	Mean of Membership Function	Sequence
15	A4B3C2	66.240 ± 0.430	7.538 ± 0.024 a	6.378 ± 0.004 b	8.833 ± 0.088 c	4.225 ± 0.028 a	0.959	1
14	A4B2C3	52.953 ± 0.454	5.111 ± 0.016 e	6.230 ± 0.004 c	7.800 ± 0.100 e	3.299 ± 0.026 b	0.747	2
3	A1B3C3	47.290 ± 0.337	6.885 ± 0.015 b	4.371 ± 0.002 j	10.200 ± 0.058 a	2.067 ± 0.014 e	0.720	3
4	A1B4C4	52.780 ± 0.284	5.726 ± 0.014 d	5.089 ± 0.012 f	8.567 ± 0.088 d	2.686 ± 0.009 c	0.712	4
2	A1B2C2	36.400 ± 0.243	3.747 ± 0.018 g	6.444 ± 0.013 a	8.900 ± 0.058 c	2.346 ± 0.014 d	0.635	5
8	A2B4C3	42.300 ± 0.560	6.860 ± 0.016 b	4.463 ± 0.006 i	6.633 ± 0.033 f	1.888 ± 0.024 f	0.596	6
16	A4B4C1	41.653 ± 0.269	5.557 ± 0.030 d	3.792 ± 0.003 k	6.433 ± 0.033 g	1.579 ± 0.010 g	0.500	7
7	A2B3C4	32.303 ± 0.527	6.097 ± 0.050 c	4.530 ± 0.002 h	9.100 ± 0.115 b	1.464 ± 0.025 h	0.466	8
12	A3B4C2	24.667 ± 0.333	4.056 ± 0.056 f	5.180 ± 0.004 e	5.867 ± 0.033 i	1.278 ± 0.018 i	0.416	9
6	A2B2C1	31.550 ± 0.165	3.103 ± 0.052 i	4.680 ± 0.003 g	5.233 ± 0.067 k	1.476 ± 0.009 h	0.377	10
5	A2B1C2	16.910 ± 0.240	2.361 ± 0.073 kl	5.277 ± 0.006 d	6.233 ± 0.033 h	0.892 ± 0.012 k	0.325	11
13	A4B1C4	30.060 ± 0.394	3.411 ± 0.048 h	3.535 ± 0.022 l	5.367 ± 0.033 k	1.063 ± 0.012 j	0.319	12
11	A3B3C1	15.723 ± 0.182	3.361 ± 0.073 h	2.273 ± 0.002 o	6.300 ± 0.058 gh	0.357 ± 0.004 m	0.207	13
10	A3B2C4	25.997 ± 0.508	2.533 ± 0.033 jk	2.045 ± 0.003 p	5.533 ± 0.033 j	0.532 ± 0.011 l	0.191	14
1	A1B1C1	11.870 ± 0.406	2.722 ± 0.147 j	2.852 ± 0.008 n	4.800 ± 0.058 l	0.339 ± 0.011 m	0.152	15
9	A3B1C3	8.057 ± 0.471	2.167 ± 0.167 l	3.348 ± 0.004 m	4.567 ± 0.033 m	0.270 ± 0.015 n	0.125	16
17	CK	10.413 ± 2.309	1.833 ± 0.167 m	1.628 ± 0.015 q	3.067 ± 0.088 n	0.169 ± 0.003 o	0.008	17

Note: The data in the table are the average values of three repetitions ± the standard error; Different letters in the same column indicate significant differences between treatments (*p* < 0.05), while the same letter indicates no significant difference between them (*p* > 0.05).

## Data Availability

The basic data for this article can be found in the article. However, some data is currently not shared and is also part of ongoing research. If necessary, it can be obtained from the corresponding author upon reasonable request.
